# Time trends in Surgical Site Infection rates in a Tunisian university hospital

**DOI:** 10.1093/eurpub/ckac131.386

**Published:** 2022-10-25

**Authors:** H Ghali, S Bhiri, A Ben Cheikh, R Bannour, S Khefacha, M Ben Rejeb, H Said Latiri

**Affiliations:** 1 Department of Prevention and Security of Care, Sahloul University Hospital, Sousse, Tunisia; 2 Faculty of Medicine of Sousse, University of Sousse, Sousse, Tunisia; 3 LR20SP06, Emerging Bacterial Resistance in Hospitals Veterinarians and the Environment and Security of Care, Tunisia

## Abstract

**Background:**

Surgical site infections (SSIs) are the most common hospital-acquired infections (HAIs) in low- and middle-income countries. Many reports have shown that surveillance and management of factors associated with SSI decreased rates and improved overall outcomes.This study aimed to appraise the prevalence trend and risk factirs of SSIs during 10-year period (2012 - 2021) in a Tunisian university hospital.

**Methods:**

The SSI surveillance module is based on the National Healthcare Safety Network (NHSN), Centers for Disease Control and Prevention (CDC). For the current study, data collected over ten years through point prevalence surveys were analyzed. Univariate and multivariate logistic analysis were used to identify SSI risk factors.

**Results:**

Overall, 2957 patients were observed; the mean age was 48.4 ± 23.5 years and 57.2% were male. We identified 289 infected patients (9.8%) and 319 HAIs (10.8%). SSIs were found in 21.6% of cases.The prevalence of SSI decreased from 27.9% in 2012 to 21.6% in 2021. However, this decrease was not statistically significant. The majority of the positive cultures were Staphylococcus aureus (14.3%) followed by Escherichia Coli (11.1%) and Klebsiella pneumoniae (9.5%). Antimicrobial resistance was found in 17.5% of cases. Univariable analysis found that lenght of stay (p < 10-3), obesity (p = 0.047), the use of antibiotic treatment in 6 months (p = 0.002), and the use of central line (p < 10-3) were associated with SSI. Independent risk factors significantly associated with SSIs were length of stay (aOR=8.6), the use of central line (aOR=3), and the use of antibiotic treatment in 6 months (aOR=2.2).

**Conclusions:**

With continuous surveillance, the prevalence of SSIs decreased. In Sahloul university hospital, there has been a strengthening of the application of hygiene standard precautions during the two last years, and more particularly the respect of hand hygiene, combined with continued inpatient antimicrobial stewardship programs.

**Key messages:**

• Active surveillance and management of factors associated with surgical site infection (SSI) decreased the incidence and improved overall outcomes.

• With continuous surveillance, the prevalence of SSIs decreased over the 10-year study period.

